# Capturing Conformational States in Proteins Using Sparse Paramagnetic NMR Data

**DOI:** 10.1371/journal.pone.0127053

**Published:** 2015-05-18

**Authors:** Kala Bharath Pilla, Julia Koehler Leman, Gottfried Otting, Thomas Huber

**Affiliations:** 1 Research School of Chemistry, Australian National University, Canberra, ACT 2601, Australia; 2 Department of Chemical and Biomolecular Engineering, Johns Hopkins University, Baltimore, MD, 21218, United States of America; Oak Ridge National Laboratory, UNITED STATES

## Abstract

Capturing conformational changes in proteins or protein-protein complexes is a challenge for both experimentalists and computational biologists. Solution nuclear magnetic resonance (NMR) is unique in that it permits structural studies of proteins under greatly varying conditions, and thus allows us to monitor induced structural changes. Paramagnetic effects are increasingly used to study protein structures as they give ready access to rich structural information of orientation and long-range distance restraints from the NMR signals of backbone amides, and reliable methods have become available to tag proteins with paramagnetic metal ions site-specifically and at multiple sites. In this study, we show how sparse pseudocontact shift (PCS) data can be used to computationally model conformational states in a protein system, by first identifying core structural elements that are not affected by the environmental change, and then computationally completing the remaining structure based on experimental restraints from PCS. The approach is demonstrated on a 27 kDa two-domain NS2B-NS3 protease system of the dengue virus serotype 2, for which distinct closed and open conformational states have been observed in crystal structures. By changing the input PCS data, the observed conformational states in the dengue virus protease are reproduced without modifying the computational procedure. This data driven Rosetta protocol enables identification of conformational states of a protein system, which are otherwise difficult to obtain either experimentally or computationally.

## Introduction

The ability to change conformation is critical for proteins to regulate binding and function. Large conformational changes are often observed in proteins on binding of ligands, co-factor molecules or change in physiological conditions. While mapping these structural changes is fundamental to our understanding of a protein’s mechanism of action, structural characterization of condition-induced conformational changes can be challenging as they often resist common experimental approaches. In X-ray crystallography, conformational flexibility is a severe impediment to crystallization and, if crystallization is successful, generally leads to lower data quality. In addition, successful structure determination by X-ray crystallography typically lies within a narrow range of conditions [1] and small changes, such as variations of the physiochemical environment or changing the enzyme from holo to apo form, can render structure determination impossible. Solution NMR spectroscopy in contrast offers more flexibility in measuring data in varying chemical environments. 3D structures can be determined by resolving a dense network of short-range (< 5 Å) distance restraints from nuclear Overhauser effects (NOE) in physiological, but also extreme environmental conditions[2,3]. Steric changes that cause chemical shift perturbations can also be resolved qualitatively from straightforward 2D NMR experiments [4]. However, high molecular weight proteins and protein complexes pose difficulties because they often produce poor solution NMR spectra due to overlapping peaks or broad and missing signals.

More recently, alternative computational/experimental hybrid approaches have been developed that use sparse paramagnetic NMR measurements to generate 3D atomic models of proteins or protein complexes [5,6]. Paramagnetic ions in protein samples induce significant effects in NMR spectra due to the 650-fold larger magnetic moment of a free electron compared to a proton. While the effects of paramagnetism on nuclear magnetic resonance have been studied since the very beginning of NMR spectroscopy [7], only recent advances in site specific labeling of proteins with chemical lanthanide tags have made the approach more generally applicable (see reviews [8–12]).

Paramagnetic metal ions with a non-vanishing anisotropic magnetic susceptibility tensor (Δχ-tensor) lead to weak molecular alignment with the magnetic field, resulting in observable residual dipolar couplings (RDC) which give information about the orientation of spin pairs with respect to the molecular alignment tensor in a distance independent manner. Sparse RDC datasets have been used to guide the conformational search in Rosetta *de novo* structure determination by fragment assembly [13]. Furthermore, when sparse RDC data are combined with sparse backbone NOE data, proteins up to 30 kDa can be modeled in Rosetta with a resolution that approaches that of the native structure [13]. RDCs combined with paramagnetic relaxation enhancement (PRE) measurements have also been shown to resolve the structures of α-helical proteins using a molecular fragment replacement method [14].

Among the paramagnetic effects in NMR spectroscopy, pseudocontact shifts (PCS) are arguably the most potent for structure elucidation. PCSs are manifested as simple differences of chemical shifts between a protein’s paramagnetic and diamagnetic states, and therefore can be measured with much higher sensitivity and accuracy than NOEs, RDCs, and PREs. The PCS of a nuclear spin induced by a paramagnetic center is directly related to structural information by the following equation:

$$\delta^{PCS}=\frac{1}{12\pi r^{3}}\left[\Delta \chi_{ax}\left(3\cos^{2}\theta -1\right)+\frac{3}{2}\Delta \chi_{rh}\left(\sin^{2}\theta \cos2\varphi \right)\right]$$ (1)

where δ^PCS^ is measured in ppm, *r*, θ, and φ are the polar coordinates of the nuclear spin with respect to the principal axes of the Δχ-tensor, and Δχ_ax_ and Δχ_rh_ define the axial and rhombic components, respectively, of the Δχ-tensor [15]. Most importantly, PCSs are both distance and orientation dependent and can be measured for spins over a maximum range of 80 Å (within 40 Å from the paramagnetic center) [16]. These long-range distance restraints make PCSs exquisitely useful for protein structure determination with Rosetta, as they provide reliable information on the overall protein fold topology while Rosetta is proven to generate high resolution structures once sampling can be concentrated close to the native structure [17].

Previously, we have shown that structures of small proteins can be calculated using backbone amide PCS data from a single metal center when used as restraints within Rosetta [18]. Furthermore, we have extended our approach to use PCS data from multiple metal binding tags by treating different metal centers in a manner analogous to GPS satellites, which improves both sampling efficiency and accuracy. The PCSs of a spin determined from three or more metal centers can be used to accurately restrain the coordinates in 3D space, and we have integrated this method, dubbed GPS-Rosetta, into the Rosetta software suite [19].

So far our approach has been limited to structure calculations of monomeric proteins with less than 150 residues. Success in calculating larger protein structures by PCSs alone depends mostly on computational time and whether homologous protein structures exist to improve the Rosetta fragment libraries. In the case of large proteins, the conformational search space is simply too vast and attaining near-native models in finite time is often impossible even with the availability of overlapping sets of PCS data from multiple paramagnetic metal centers. The conformational search space is greatly reduced, however, if a part of the structure of a protein is already known in advance. In this situation, the Δχ-tensors can be defined by the PCSs from the structure of the known part and the Δχ-tensors will turn the remaining PCSs into precise restraints for conformational sampling of the structurally unknown part. This approach is tested in the present work designed to capture the conformational states of the 27 kDa dengue virus serotype 2 (DENV) NS2B-NS3 protease.

**Fig 1:**
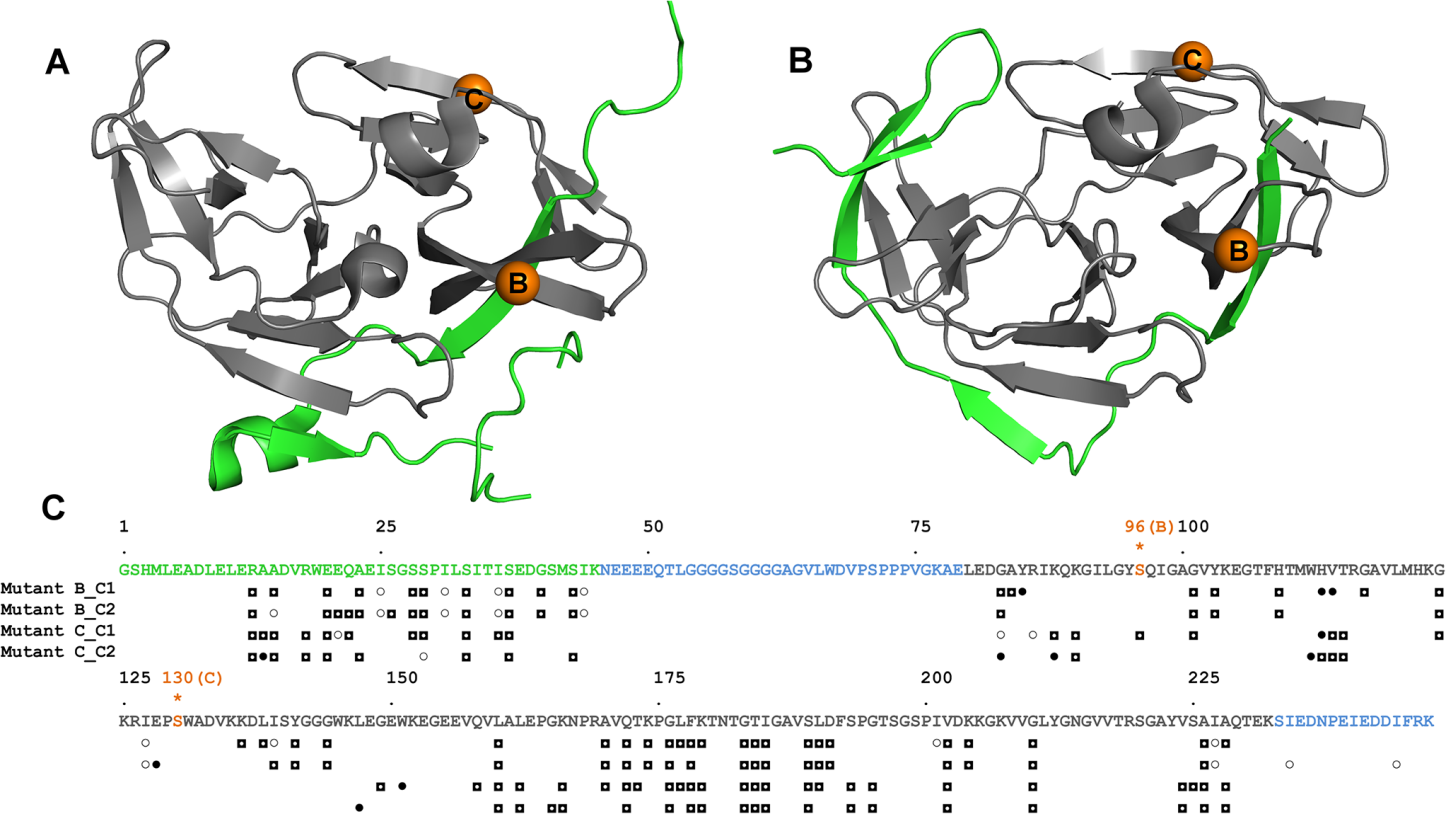
Crystal structures of DENV NS2B-NS3pro and overview of PCSs measured versus the amino acid sequence. (A) Open state as observed in the ligand-free conformation in the crystal structure 2FOM [26]. NS2B is shown in green and NS3pro is shown in grey. The orange balls identify the locations of residues 34 and 68. We refer to the mutants S34C and S68C as mutants B and C, respectively [23]. To induce PCSs in the protein, the mutants B and C were reacted with a single lanthanide binding tag to form a disulfide bond with either of the cysteine residues at these sites. (B) Closed state as observed in the crystal structure 3U1I [22] with the same color coding as in (A). The closed conformation is presumed to represent the enzymatically active state. (C) Summary of the experimental PCSs. C1 and C2 denote two different lanthanide binding tags used. They differ only in chirality [23,28]. Open circles, filled circles, and boxes identify the residues for which PCSs were observed with Tb^3+^, Tm^3+^, or both Tb^3+^ and Tm^3+^, respectively. The residue numbering used is shown at the top of the amino acid sequence. In this numbering scheme, the mutants B and C are at residues 94 and 129 (highlighted in orange). The green and grey characters identify, respectively, the NS2B and NS3pro segments for which coordinates are reported in the crystal structures (PDB ID: 2FOM, 3U1I). Sequence segments for which no electron density was observed, are highlighted in blue. These parts are probably flexible [25–27].

The active form of the DENV NS2B-NS3 protease consists of two domains, where the N-terminal part of the non-structural protein 3 (NS3) encodes a serine protease (NS3pro), and a segment of 40 residues from the non-structural protein 2 (NS2B) forms the co-factor domain (Fig 1). For both functional [20] and structural studies [21–23], NS2B was fused to the N-terminus of the NS3pro via a nine residue (Gly_4_-Ser-Gly_4_) linker. The presence of NS2B in the 247 amino acid residue fusion construct increases the proteolytic activity 3300–7600 fold [24]. In structural studies, the protease was found to exhibit two distinct conformations referred to as 'open' and 'closed' states. In the absence of inhibitors, crystal structures of the dengue virus NS2B-NS3 protease and the closely related corresponding protease from West Nile virus show NS2B adopting a conformation that positions it far from the active site ('open state'), except for the N-terminal segment (residues 1–15) of NS2B which forms part of the N-terminal β-barrel of NS3pro as shown in Fig 1A [25–27]. In contrast, crystal structures with an inhibitor in the active site display a significant conformational change, resulting in a structure where NS2B is wrapped around NS3pro forming a β-hairpin near the active site and directly interacting with the substrate ('closed state') as shown in Fig 1B [22]. In this condition-induced conformational rearrangement, the coordinates of NS3pro remain largely unchanged while the structure and orientation of NS2B with respect to NS3pro change dramatically and need to be determined. Instead of determining the structure of the entire NS2B-NS3pro construct *de novo*, we approached this problem by calculating the spatial coordinates of NS2B alone in a structure completion manner using GPS-Rosetta protocol. This approach not only presents a new method for data assisted structure completion but also calculates the structure of NS2B from sparse experimental PCS data.

## Experiments and Methods

### PCS Data

#### Experimental PCS data in the closed state

Previously published PCS data for the closed state conformation of the dengue viral protease [23] were used as input for GPS-Rosetta. In the sequence numbering of the present work (Fig 1C), the available PCS data pertained to three different cysteine mutants, Ala15Cys (mutant A), Ser94Cys (mutant B), and Ser129Cys (mutant C). Each of the mutants was independently ligated with either of two different lanthanide-carrying chemical tags, C1 and C2 [28], by formation of a disulfide bridge with the introduced cysteine residue. Each chemical tag was loaded with either Tb^3+^, Tm^3+^, and Y^3+^, where Y^3+^ served as diamagnetic reference. The dataset from mutant A was omitted purposely as Ala 15 is located in NS2B. The PCS data of the final input was thus derived from mutants B and C, comprising a total of 334 PCSs from four metal centers (eight paramagnetic samples), of which 94 are in NS2B and 240 are in NS3pro. Fig 1C shows a schematic overview of the PCS data from each of the mutants.

#### Calculated PCS data in the open state

The ligand-free open state conformation lacks experimental PCS data and the X-ray crystal structure (PDBID: 2FOM) is missing electron density for a number of residues in NS2B, the Gly_4_-Ser-Gly_4_ linker, and NS3pro, suggesting flexibility in these regions [26]. To obtain a full set of coordinates we used the X-ray crystal structure as a template and computed missing coordinates of the open state structure with the program Modeller [29]. The new model has the same sequence length as the closed state construct. PCS data were then generated for the new open state model using the experimentally determined Δχ-tensors reported for mutants B and C of the closed state structure [23]. No uncertainties were added to the generated datasets to avoid losing the information contained in very small PCSs. The generated datasets comprised PCSs only for those residues, for which experimental PCSs were available in closed state. The final eight datasets for the open state were thus of the same size as the experimental data of the closed state, comprising 94 PCSs in NS2B and 240 PCSs in NS3pro from four different metal centers.

### GPS-Rosetta calculations

The Rosetta *ab initio* sampling algorithm used Monte-Carlo assembly of nine and three residue fragments which were generated using sequence information from the target protein [30]. The fragment libraries for NS2B-NS3pro were generated on the Robetta server [31] with additional input of diamagnetic chemical shifts for backbone atoms of the closed structure [23] to improve the quality of the generated fragments [32]. The libraries did purposefully include fragments from homologous structures, the closed state structure from West Nile virus (PDBID: 2FP7) and the open state structure from DENV (PDBID: 2FOM) [26], but did not contain the closed state native structure of the DENV NS2B-NS3pro.

**Fig 2:**
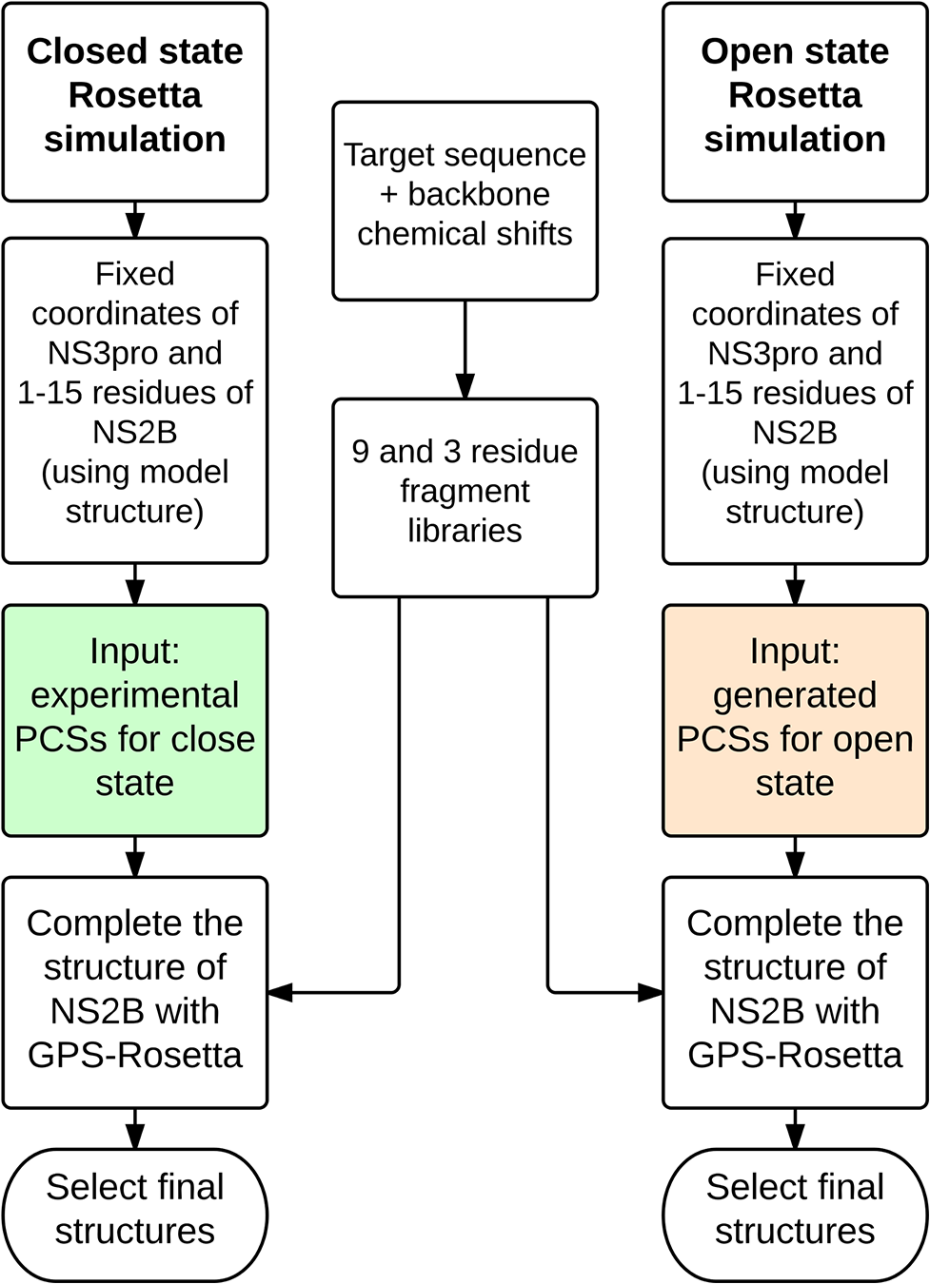
Flow chart of calculations. The flow chart outlines the simulations performed to determine the orientation of NS2B with respect to NS3pro, using different PCS datasets for closed and open conformations.

The structure of NS2B, in particular of residues 16–44, is crucial for understanding the active form of the protease. Two simulations, which sampled possible conformations, were carried out independently, but using identical computational setup. Fig 2 outlines the structural calculations in a flow chart. The computations on the closed conformation of NS2B were carried out with experimental PCS data as input and the open conformation was calculated with ersatz PCS data.

All Δχ-tensors for the individual metal centers were optimized simultaneously during the Rosetta simulation. The fit quality for each lanthanide tag site *k* was scored as

$$s_{k}=Rc\overset{m}{\underset{q=1}{\sum }}\sqrt{\overset{n_{pcs}}{\underset{p=1}{\sum }}{\left(PCS_{calc}^{pq}-PCS_{exp}^{pq}\right)}^{2}}$$ (2)

where *m* is the number of PCS datasets (one dataset per metal ion) and *n*_*pcs*_ is the number of PCSs in the dataset. *Rc* is a unity constant in units of $\frac{REU}{ppm}$ to convert PCS root-mean-square deviations to Rosetta energy units (REU).

PCS fit quality scores for each of the metal centers were independently weighted relative to the Rosetta low-resolution scoring function and the total weighted sum score S_total_ was added to the low-resolution energy function of Rosetta:

$$S_{total}=\overset{ntag}{\underset{k=1}{\sum }}s_{k}.w_{k}$$ (3)

The weighting factor (*w)* for each of the four metal centers was calculated by generating 1000 structures without PCS restraints, followed by calculation of *w* using

$$w=\left(\frac{a_{high}-a_{low}}{c_{high}-c_{low}}\right)\;/\;4$$ (4)

where a_high_ and a_low_ are the averages of the highest and lowest 10% of the values of the Rosetta *ab inito* score and c_high_ and c_low_ are the averages of the highest and lowest 10% of the PCS energy score value obtained by rescoring 1000 decoys with a unity PCS weighting factor. For PCS energy rescoring, the weighting factors were set to unity.

The conformational sampling was carried out independently for the closed and open states, and in both simulations the coordinates of NS3pro were kept rigid along with the coordinates of the first 15 residues of NS2B. To achieve this, starting models were provided as input to GPS-Rosetta. The structure derived from NMR structural studies [23] was used as a reference for the closed state, because the only available crystal structure 3U1I [22] is from dengue serotype-3 which possesses as little as 66% sequence identity to the target protein from dengue serotype-2. For the open state simulation, the homology model generated from the crystal structure 2FOM [26] was used. Because the coordinates in the NS3pro domain are fixed, they are also excluded in RMSD calculations. Including the larger fixed NS3pro domain in the analysis would only dilute the effect of NS2B changes and lead to reporting unreasonably small RMSD values.

A total of 10,000 all-atom models were generated in each of the closed and open state Rosetta calculations. The constraints fixing the atomic coordinates of NS3pro led to chain breaks in the NS2B segment in nearly 50% of the sampled models and these models were not used in further analyses. From the remaining models, unstructured regions at the C-terminus of NS3pro and the glycine linker (highlighted in blue in Fig 1C) were deleted to obtain energy scores for the structured parts of NS2B and NS3pro. The models were rescored with both the Rosetta all-atom energy function and weighted PCS energy, and the structures with lowest combined energy scores were selected as final structures.

The current GPS-Rosetta algorithm took about 5,000 CPU hours to sample 20,000 all-atom models and the program parallelizes nearly linearly on conventional computer clusters, offering rapid structural characterization. The algorithm is incorporated into the Rosetta software package and is available for download at https://www.rosettacommons.org. The protocol was independently tested by a member of RosettaCommons and the experimental and calculated PCS datasets along with all protocols are made publicly available in the protocol capture directory of the current Rosetta release.

## Results and Discussion

### Solution structure of NS2B in complex with NS3pro

**Fig 3:**
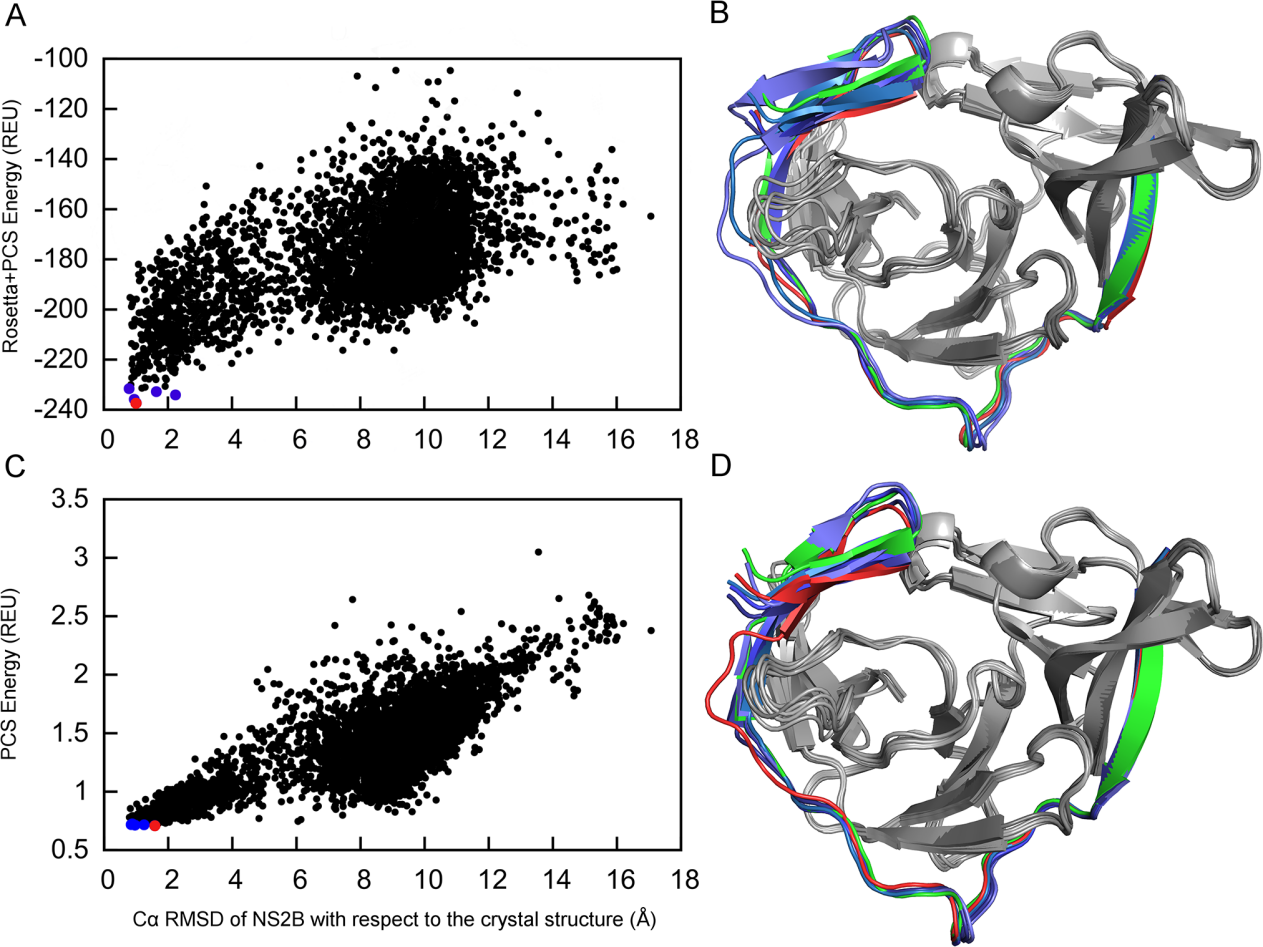
Closed state conformation of NS2B determined by GPS-Rosetta. (A) Scatter plot of 5,000 all-atom structures showing their combined score of weighted PCS + Rosetta energy versus the Cα RMSD of NS2B relative to the crystal structure in the closed conformation (PDB ID 3U1I). The RMSD was calculated for NS2B only (residues 50–87 of chain A in 3U1I). The conformation selected as the best structure (red point) has the lowest combined energy score and is referred to as the “closed GPS-Rosetta structure”. The four next-lowest combined score structures are represented by blue points. (B) Comparison between GPS-Rosetta structures and the crystal structure. The closed GPS-Rosetta structure is shown in red (NS2B) and grey (NS3pro) and the crystal structure 3U1I is shown in green (NS2B) and grey (NS3pro). The Cα RMSD of NS2B in the closed GPS-Rosetta model is 1.0 Å relative to the crystal structure. The NS2B segments of the next four lowest-energy structures have RMSDs ranging from 0.7 to 2.2 Å and are displayed in different shades of blue. The superimposition shown in the figure used the Cα atoms of NS2B only. (C) Same as (A), except that scoring used PCSs only. (D) Same as (B), except using the structures with the lowest PCS scores identified in (C). In all five models, NS2B has a Cα RMSD between 0.8 and 1.6 Å relative to the NS2B part in the crystal structure.

The structure determination of NS2B was aided by PCSs from four different metal centers and the model quality was evaluated by energetic criteria as well as by the overall agreement with the experimental PCS data. Fig 3 shows the combined PCS and Rosetta energy for each of the models relative to the RMSD with respect to the closed state in the crystal structure 3U1I [31]. The structure with the lowest combined Rosetta and PCS energy score was selected as the best structure. It is represented by a red point in Fig 3A. Following superimposition of the best structure on the crystal structure 3U1I resulted in a Cα RMSD value of 1.0 Å for NS2B (shown in red in Fig 3B). In the following, we refer to this structure as the closed GPS-Rosetta structure. In every one of the best five structures, the C-terminal segment of NS2B formed the β-hairpin motif near the active site detected by the crystal structure 3U1I. The excellent structural agreement with the crystal structure confirms that the binding mode of NS2B to NS3pro is the same in solution as in the crystal structure. This structural conservation is of particular importance as the PCSs were measured in complex with a different low-molecular weight inhibitor [23] than the crystal structure [22]. Our computations thus suggest that the closed GPS-Rosetta structure is energetically favorable also in the absence of an inhibitor. This result is supported by the observation that samples, in which the covalent link between NS2B and NS3pro is severed by proteolysis, predominantly assume the closed conformation [33,34].

**Fig 4:**
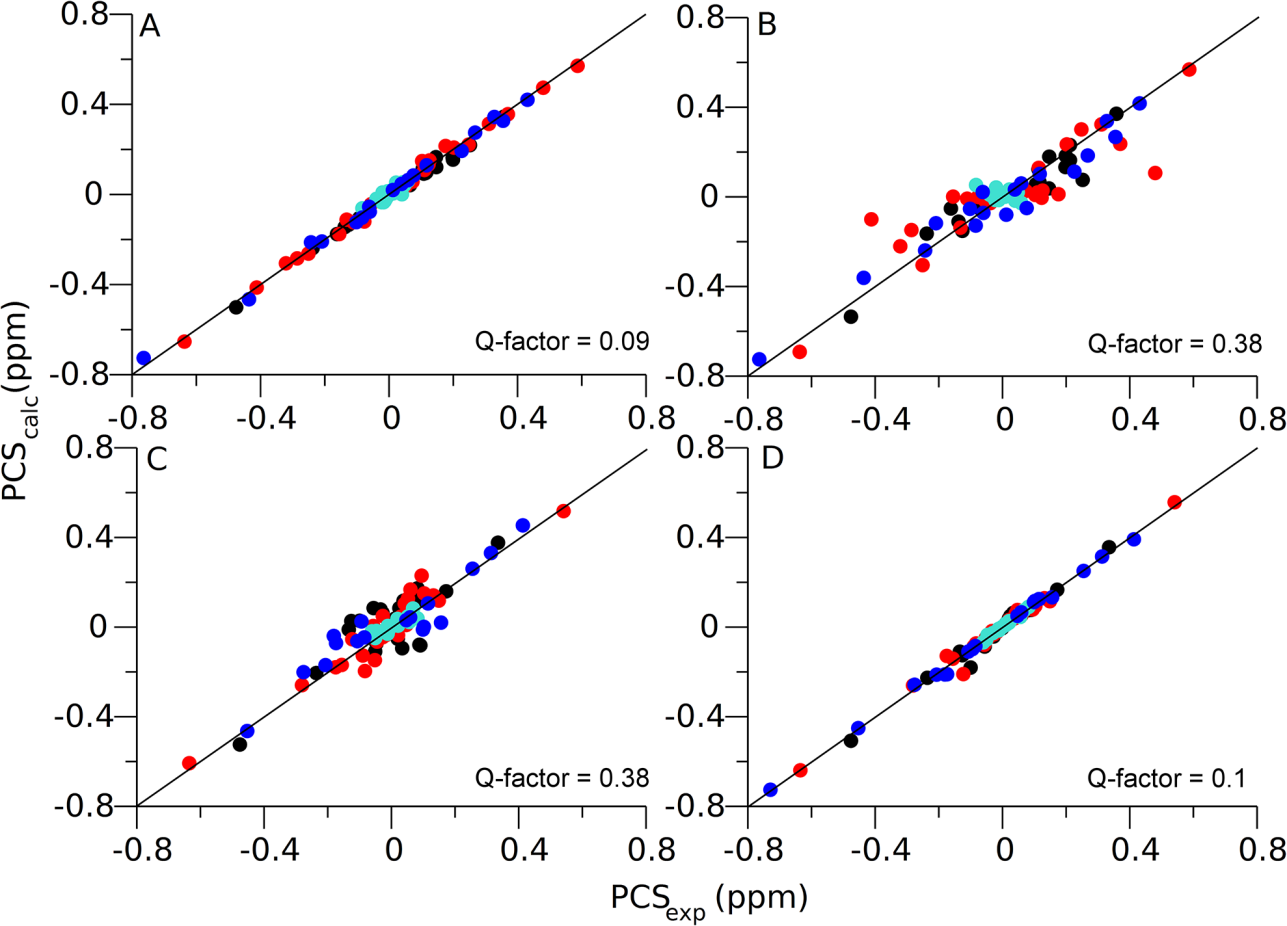
Correlations between experimental and back-calculated PCSs fitted to the closed and open GPS-Rosetta conformations. To illustrate the information content associated with the PCSs, experimental PCSs measured for the closed state and PCSs generated for the open state (both reported as PCS_exp_) were used to fit Δχ-tensors to either the closed or open GPS-Rosetta structures. Subsequently, the Δχ-tensors were used to back-calculate PCS_calc_ values. Data from Mutant B_C1, Mutant B_C2, Mutant C_C1, and Mutant C_C2 are represented in black, red, cyan, and blue, respectively. Q-factor calculations used the PCSs of backbone amide protons of NS2B only. The correlation plots were produced for all four possible combinations: (A) experimental PCSs for the closed state and closed GPS-Rosetta structure; (B) experimental PCSs of the closed state and open GPS-Rosetta structure; (C) PCSs generated for the open state and closed GPS-Rosetta structure; (D) PCSs generated for the open state and open GPS-Rosetta structure.

The agreement of the model with experimental PCS data is validated by the good agreement between input PCS data and PCS data back-calculated using the best-fitting Δχ-tensors, which is reflected in low quality factors (Q-factors) [35]. Table 1 reports the magnitudes of the fitted Δχ-tensors for each of eight metal centers along with the Q-factors. All Q-factors are less than 19% for the combined NS2B-NS3pro structure. The correlation plot between experimental and back-calculated PCSs of NS2B in the closed GPS-Rosetta structure shows that all PCS values are reproduced by the model within the experimental error of 0.05 ppm (Fig 4A).

**Table 1:** Effective Δχ-tensor parameters of the eight different lanthanide tags for both closed and open GPS-Rosetta models.

Closed GPS-Rosetta structure				
Mutant	Ln^3+^	Δχ_ax_ ^a^	Δχ_rh_ ^a^	Q-factor
B_C1	Tb^3+^	-12.0	-4.3	0.10
	Tm^3+^	8.5	2.2	0.09
B_C2	Tb^3+^	-14.8	-3.8	0.07
	Tm^3+^	13.0	2.7	0.06
C_C1	Tb^3+^	21.5	11.0	0.19
	Tm^3+^	-17.0	-8.8	0.18
C_C2	Tb^3+^	-27.0	-6.5	0.10
	Tm^3+^	15.3	2.7	0.08
Open GPS-Rosetta structure				
B_C1	Tb^3+^	-10.0	-2.5	0.09
	Tm^3+^	6.7	1.4	0.07
B_C2	Tb^3+^	-15.5	-5.8	0.07
	Tm^3+^	13.0	4.3	0.05
C_C1	Tb^3+^	21.4	10.2	0.03
	Tm^3+^	-16.4	-5.2	0.04
C_C2	Tb^3+^	-17.6	-3.0	0.05
	Tm^3+^	9.4	3.3	0.06

^a^The axial and rhombic components of the Δχ-tensors are given in 10^–32^ m^3^.

The quality factors (Q) were calculated as the root-mean-square deviation between experimental and back-calculated PCSs divided by the root-mean-square of the experimental PCSs. The Q-factor calculations used all available PCSs of backbone amide protons of NS2B and NS3pro.

### Capacity to identify native-like structures

The combined Rosetta and PCS energy clearly distinguishes native conformations from the pool of structural decoys but, at the same time, the decoys that are close to the native structure (RMSD < 2 Å) span a relatively broad range of energy values from -240 to -180 REU (Fig 3A). We attribute this spread to close packing interactions in Rosetta's all-atom ‘Relax’ energy function, which is highly sensitive to minor perturbations in coordinates. In this situation, the PCS energy score, which shows the extent to which structures agree with the experimental data, provides a particularly useful measure for selecting structures without the influence of sensitive short-range energies. Fig 3C shows the PCS energy score versus the Cα RMSD of NS2B relative to the crystal structure for all models. The PCS energy is more strongly funneled towards the native structure than the combined PCS and Rosetta energy. The superimposition of the NS2B segments in the best five structures (red and blue points in Fig 3A and 3C) onto the NS2B coordinates of the closed state crystal structure 3U1I produced Cα RMSDs of less than 2.2 Å using the combined Rosetta and PCS energy and less than 1.6 Å using only the PCS energy (Fig 3B and 3D).

The ligand-free open state conformation obtained from X-ray crystallography (PDB ID 2FOM [26]) shows a very different conformation to that of the ligand bound state (Fig 1A and 1B), but experimental PCS data are not available for the open state reported by the crystal structure 2FOM. Therefore, we calculated PCSs for a model structure of the open conformation, using the Δχ-tensors reported for the closed state.

**Fig 5:**
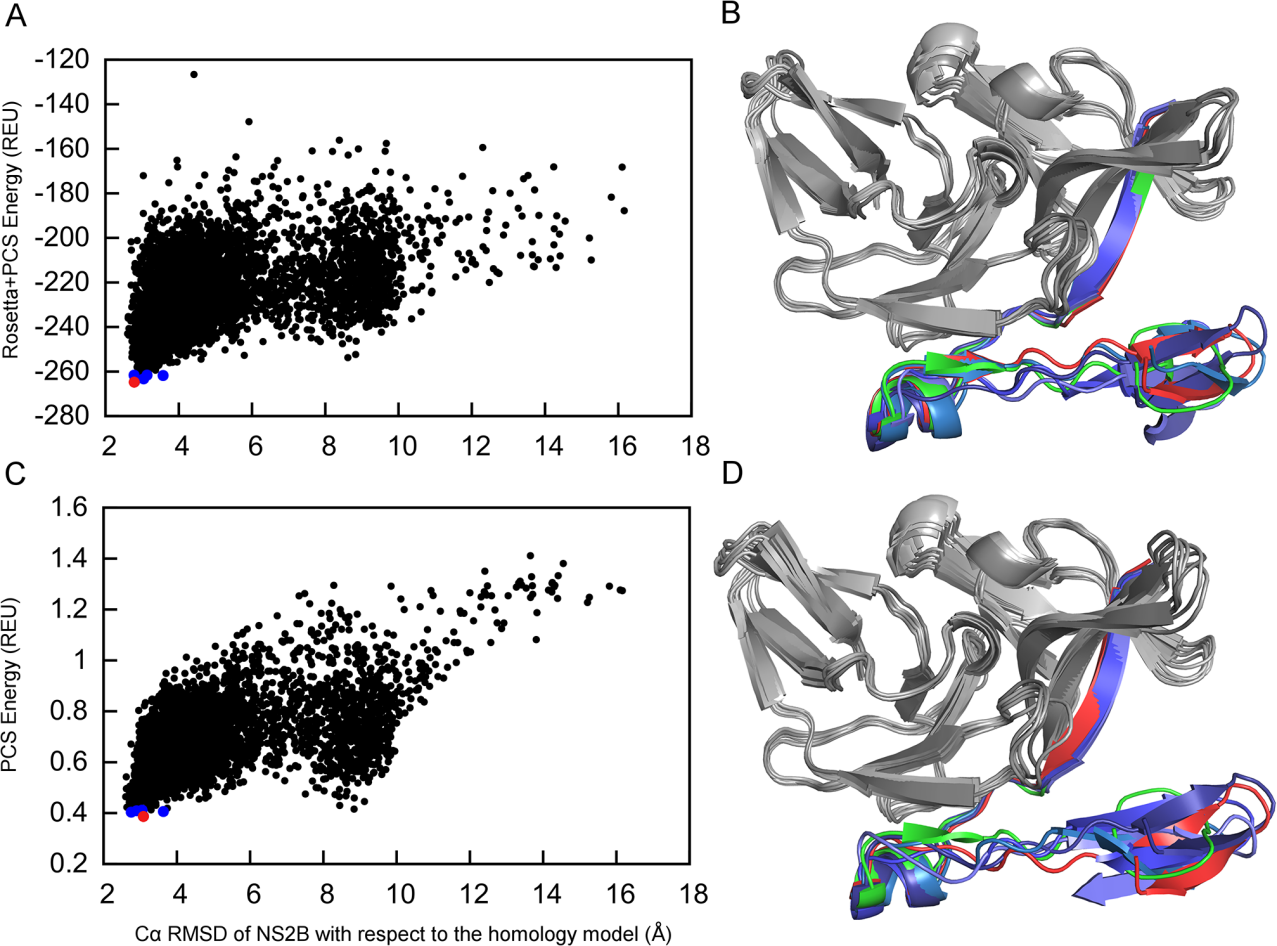
Open state conformation of NS2B generated using GPS-Rosetta. (A) Scatter plot of 5,000 all-atom structures showing their combined score of weighted PCS + Rosetta energy versus the Cα RMSD of NS2B in the homology model built on the crystal structure 2FOM of the open conformation. The final selected structure (red point) has the lowest combined energy score and is referred to as the “open GPS-Rosetta structure”. The four next-lowest combined score structures are represented by blue points. (B) Superimposition of the best GPS-Rosetta structures onto the homology model. The open GPS-Rosetta structure is shown in red (NS2B) and grey (NS3pro) and the homology model in green (NS2B) and grey (NS3pro). The Cα RMSD of NS2B in the open GPS-Rosetta model is 2.7 Å relative to the homology model. The NS2B segments of the next four lowest-energy structures have RMSDs ranging from 2.7 to 3.1 Å and are displayed in different shades of blue. The superimposition shown in the figure used the Cα atoms of NS2B only. (C) Same as (A), except that scoring used PCSs only. (D) Same as (B), except using the structures with the lowest PCS scores identified in (C). In all five models, NS2B has a Cα RMSD between 2.7 and 3.6 Å relative to the NS2B part in the homology model.

For a stringent test, we applied the proposed method to calculate structures of the open state using the calculated PCS input data without changing the amino acid sequence, computational procedure or fragment libraries that were used to calculate the closed state structure. Fig 5 shows the Rosetta and PCS energy scores for each of the generated models with respect to the Cα RMSD of NS2B in the open state. The model with the lowest combined Rosetta and PCS energy score was again selected as the best model structure and is referred to in the following as the open GPS-Rosetta structure. The NS2B domain of this structure has a Cα RMSD of 2.7 Å relative to the NS2B domain of the reference homology model. As in the case of the closed GPS-Rosetta structure, the agreement of the open GPS-Rosetta structure with the input PCS data was confirmed by Δχ-tensor parameter fits and Q-factors (Table 1). The Q-factors calculated for the PCSs in NS2B and NS3pro were less than 9% for all tags and lanthanides, indicating excellent agreement between back-calculated and input PCS data. The agreement is confirmed by correlation plots between back-calculated and input PCS data using only NS2B data (Fig 4D). The four next-best models had Cα RMSDs ranging from 2.7 to 3.1 Å (Fig 5A) and superimposed reasonably closely on the open GPS-Rosetta structure and the crystal structure 2FOM (Fig 5B). Although the Cα RMSD of 2.7 Å between the open GPS-Rosetta model and the crystal structure appears to be relatively high, we note that the problem is challenging as the location of the C-terminal segment of NS2B is much less restrained by contacts with NS3pro than in the closed conformation. Therefore, the failure to generate structures with lower RMSDs may be attributed to insufficient sampling of NS2B conformations in the open state. Furthermore, the Rosetta energy function favored the formation of regular secondary structure elements, generating a β-hairpin motif for residues 31–44 which is not found in the crystal structure 2FOM but exists in the closed state crystal structure 3U1I. All the selected open state models exhibited the β-hairpin motif (Fig 5B). Interestingly, a crystal structure of an open conformation of the homologous NS2B-NS3 protease from the West Nile virus displays the β-hairpin motif of NS2B even though it is located far from NS3pro [25], confirming the propensity of this segment to form a β-hairpin independent of NS3. The open conformation of the 2FOM structure could easily be a crystallization artifact.

Sampling of the 2FOM conformation could be improved by more heavily emphasizing the PCS energy relative to the Rosetta scoring function, as in the case of the closed conformation, the PCS energy scores of models generated in the open state GPS-Rosetta simulation displayed a more prominent slope towards the native structure than the combined Rosetta and PCS energy (Fig 5C). In contrast to the situation of the closed conformation, however, the Cα RMSD values of the best five structures ranged from 2.7 to 3.6 Å, which is worse than the result obtained from the combined Rosetta and PCS energy score. All five structures exhibited the β-hairpin at the C-terminus of NS2B (Fig 5D). The same β-hairpin formed in structures from the second energy minimum observed at 9 Å RMSD in the plot of Fig 5C. While the lowest-energy structure in this second energy minimum had a Cα RMSD of 0.9 Å for the first 29 residues of NS2B, the residues 30–47 were highly disordered.

### Data-driven structure calculation

**Fig 6:**
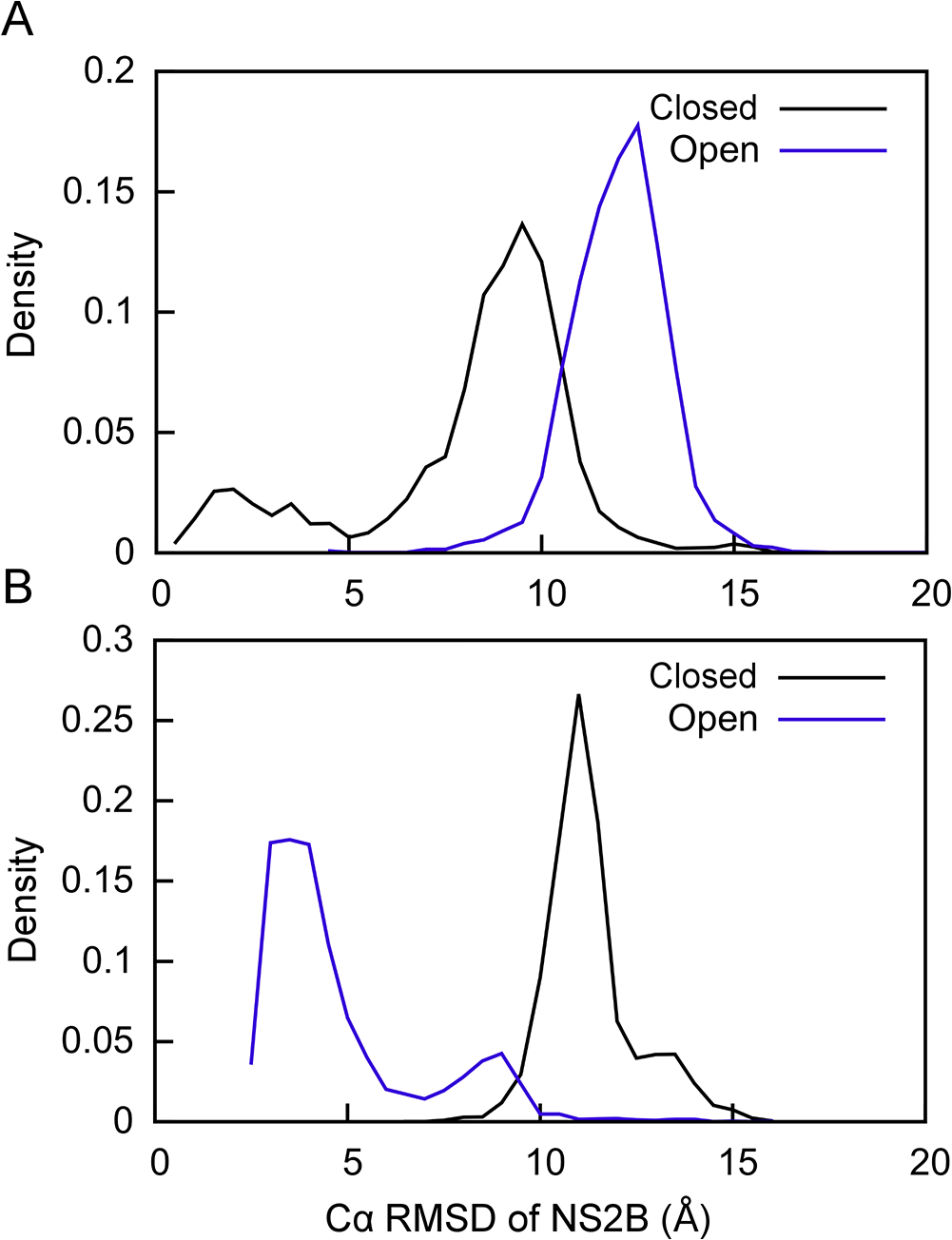
Density plots illustrating the conformational sampling bias created by PCS data. (A) Rosetta sampling density using the experimental PCSs of the closed state versus the Cα RMSD of NS2B in the closed state (PDBID 3U1I, chain A, residues 50–87) in black. The corresponding plot versus the Cα RMSD of NS2B in the open state is shown in blue. (B) Same as (A), except that the PCSs used were those calculated for the open state model.

Using identical Rosetta protocols to determine the structures of the closed and open states, either state was preferentially sampled for the two-domain dengue protease merely by altering the input PCS data. PCSs from multiple metal centers successfully bias Rosetta to sample conformational space towards structures that agree with the experimental data. This is demonstrated by the sampling density plots of Fig 6, where sampled models are compared with the two reference structures of NS2B in the closed and open states. When PCS restraints of the closed state were applied, the sampled structures were on average closer to the closed state than to the open state. The opposite was observed when PCS data from the open state were applied, illustrating the sensitivity of the conformational sampling to the experimental input data. The final structures calculated of the closed and open states also agreed very well with the input PCS data, as shown by the correlation plots (Fig 4A and 4D) and the small Q-factors for the PCSs of NS2B, which were as low as 10% (Fig 4A and 4D). In contrast, fitting Δχ-tensors to the closed GPS-Rosetta structure using the PCSs generated for open state model produced poor Q-factor (38%), and the same Q-factor (38%) was obtained by fitting Δχ-tensors to the open GPS-Rosetta structure using the PCSs of the closed state (Fig 4B and 4C).

**Fig 7:**
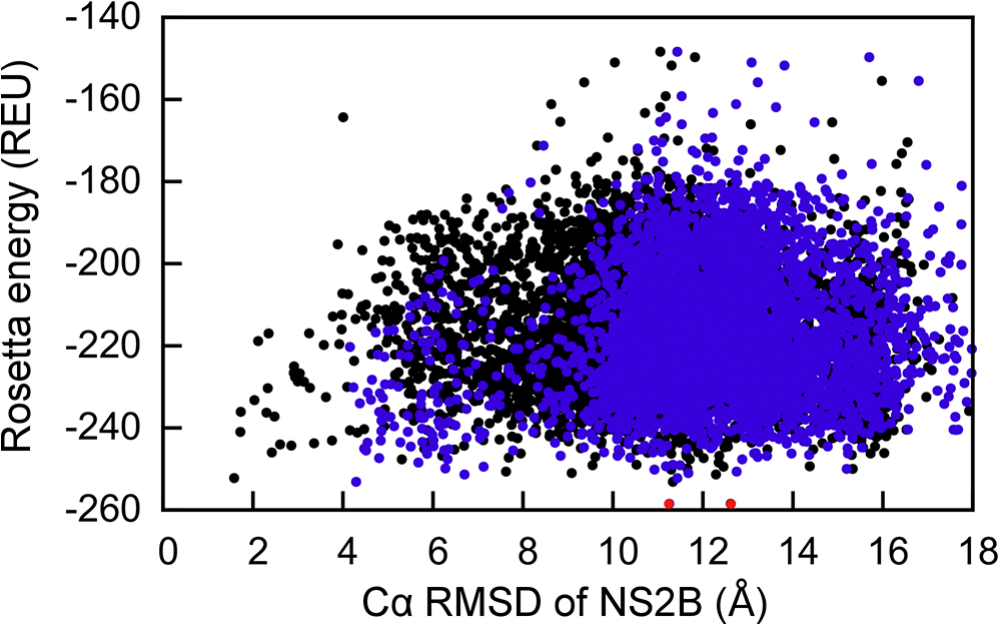
Scoring of NS2B conformations of the closed and open state by the Rosetta all-atom energy. Use of the Rosetta all-atom energy function alone fails to identify the correct structure of the closed state (black points) or the open state (blue points). The lowest-energy structures for both states are shown as red points. Their Cα RMSDs from the corresponding reference structures are about 12 Å. The overall energy landscape is more or less flat in the range from 1 to 15 Å.

The currently available computational force fields used to determine the molecular structure of proteins are less robust in discriminating between different conformational states, transient interactions, and binding modes of small molecules [6]. This was demonstrated with a negative control simulation without using PCS restraints. Identical to the simulations with PCS data, 10000 structures are generated with Rosetta. As expected, in the absence of PCSs, the all-atom energy function of Rosetta could not differentiate between the closed or open state in the pool of decoy structures. Illustrated in Fig 7, the energy landscape appeared to be flat over the RMSD range of 1–15 Å. The GPS-Rosetta protocol thus highlights the power of sparse PCS data in computational modeling, which not only directs the sampling towards the correct conformational states but also helps to identify near-native structures among a large number of structures of low Rosetta energy.

### Structural richness in sparse PCS data

PCSs offer many advantages over other NMR parameters. Most importantly, they provide unique structural information as the effect can be observed over large distances from the metal center and they are easily accessible from ^15^N-HSQC spectra that can be measured with high sensitivity. PCSs are far less laborious to measure than NOEs, as NOE measurements are less sensitive and require almost complete resonance assignments, which may be difficult to obtain for large protein systems. The DENV NS2B-NS3 protease is a classic example of a protein that produces poor NMR spectra with significant spectral overlap and signals broadened beyond detection, preventing complete resonance assignments [23].

Using different lanthanide-carrying chemical tags and in turn using different lanthanides, diverse sets of PCS data can be conveniently generated. PCSs can be measured in any physiochemical environment, in contrast to RDCs that are traditionally measured in external alignment media that often are incompatible with hydrophobic small ligands or membrane proteins [8]. As lanthanide tags that produce PCSs also generate molecular alignment and, hence, RDCs, the PCS data can in principle be complemented by RDC measurements. In addition, the paramagnetic tags produce PREs. In the present work, we did not measure RDCs because the ^15^N-HSQC cross-peaks were broad and the RDCs small, and we did not measure PREs because PREs can easily generate false positive information by intermolecular rather than intramolecular interactions.

In the context of Rosetta simulations, the long-range nature of PCS data is particularly useful to define the global fold of the protein and very sparse data sets are sufficient to compute useful structural models. In the case of DENV NS2B-NS3pro, eight paramagnetic datasets delivered no more than 49 PCSs per metal for a total of 247 residues. The coverage for NS2B was even sparser, with only 54% of the residues having PCS data from at least one metal center. The sparseness of the data did not distract, however, from the advantage of Δχ-tensors generated by different lanthanide sites. While a single PCS measured for a nuclear spin provides the information that the spin must be located on a specific PCS isosurface defined by the Δχ-tensor, a second PCS generated for the same nucleus by a lanthanide at a different site restricts the spin to lie on the intersection of the isosurfaces defined by both Δχ-tensors. A third PCS measured from a lanthanide attached at a site different from the first two would further restrict the location of the spin in space. In this way, PCSs from multiple tags can be used to position the nuclear spins in space relative to the Δχ-tensors. This technique, which is analogous to the method of finding ones location on the earth from three or more GPS satellites, is at the heart of the implemented GPS-Rosetta algorithm [19].

Despite the sparseness of the PCS data available for the DENV NS2B-NS3 protease, GPS-Rosetta faithfully determined the location and structure of NS2B relative to NS3pro with PCS data from only two different lanthanide sites. Prior to the present work, NS2B had been assumed to form the closed state based on a good match between the PCSs and a model of the closed state [23,34,36] but no alternative conformations other than the open state presented by the crystal structure 2FOM had been explored [23]. Even though the open state could not be observed in solution, there is the possibility that PCS data could be recorded in crystalline conditions. The GPS-Rosetta approach was shown to be applicable to structure calculation using PCS data recorded from micro-crystals in solid-state magic angle spinning NMR experiments [37]. The present results establish the closed state as the conformation that best combines a good fit of the PCSs with a low Rosetta energy.

Knowledge of the location of NS2B is of obvious practical importance for the rational development of inhibitors against this viral protease. Despite numerous and sustained attempts by many crystallographers, only a single structure of a dengue virus NS2B-NS3 protease (from serotype 3) has, to date, successfully been crystallized in the closed state [22]. In contrast, the described method can readily be applied for studying and identifying the conformational states of NS2B induced by inhibitors. Notably, the GPS-Rosetta algorithm does not depend on amino acid sequence information but rather determines the structure as directed by PCS data. Therefore, we expect that the GPS-Rosetta approach could readily be applied to similar systems such as the viral two-domain proteases from Japanese encephalitis virus and yellow fever virus that are homologous to DENV NS2B-NS3pro but are structurally less characterized [38,39].

## Conclusion

We have developed a new hybrid protocol that captures distinct conformational states of a protein system using PCS datasets generated from multiple metal centers to direct the conformational sampling in Rosetta. For the 27 kDa two-domain DENV NS2B-NS3 protease, the structures of closed and open conformations were readily captured using the same computational procedure but different PCS input data. The calculation of the closed state conformation, for which experimental PCS data are available, reproduced the corresponding crystal structure with remarkable accuracy. This hybrid method provides a powerful tool for detailed studies of protein conformation in difficult situations that are not amenable to established experimental or computational approaches.
